# Klotho as a biomarker of subclinical atherosclerosis in patients with moderate to severe chronic kidney disease

**DOI:** 10.1038/s41598-021-95488-4

**Published:** 2021-08-05

**Authors:** Javier Donate-Correa, Carla M. Ferri, Ernesto Martín-Núñez, Nayra Pérez-Delgado, Ainhoa González-Luis, Carmen Mora-Fernández, Juan F. Navarro-González

**Affiliations:** 1 Unidad de Investigación, Hospital Universitario Nuestra Señora de Candelaria, Santa Cruz de Tenerife, Spain; 2 GEENDIAB (Grupo Español para el estudio de la Nefropatía Diabética), Sociedad Española de Nefrología, Santander, Spain; 3 Doctoral and Graduate School, University of La Laguna, San Cristóbal de La Laguna, Tenerife Spain; 4 Clinical Analysis Service, Hospital Universitario Nuestra Señora de Candelaria, Santa Cruz de Tenerife, Spain; 5 Servicio de Nefrología, Hospital Universitario Nuestra Señora de Candelaria, Santa Cruz de Tenerife, Spain; 6 REDINREN (Red de Investigación Renal-RD16/0009/0022), Instituto de Salud Carlos III, Madrid, Spain; 7 Instituto de Tecnologías Biomédicas, Universidad de La Laguna, Santa Cruz de Tenerife, Spain

**Keywords:** Diagnostic markers, Predictive markers, Prognostic markers, Nephrology, Atherosclerosis

## Abstract

Chronic kidney disease (CKD) has been associated with a higher risk of cardiovascular disease (CVD). CKD patients present a decrease in the levels of the protein Klotho that accompanies the decrease in kidney function. This protein has been related to protective effects against CVD. However, it is unclear whether circulating Klotho, and its expression in peripheral blood cells (PBCs) are also associated with subclinical atherosclerosis in CKD. The present study aimed to study the relationship between Klotho and subclinical atherosclerosis in a population of patients with moderate to severe CKD. We determined the serum levels and gene expression in PBCs levels of Klotho and three inflammatory cytokines in 103 patients with CKD and investigated their relationship with two surrogate markers of subclinical atherosclerotis: ankle-brachial index (ABI) and carotid intima-media thickness (CIMT). Patients with subclinical atherosclerosis presented lower serum and PBCs expression levels of Klotho. Both variables were associated with the presence of subclinical atherosclerosis, being directly related with ABI and inversely with CIMT (*P* < 0.0001 for both). Multiple regression analysis demonstrated that both variables were significant determinants for ABI (adjusted R^2^ = 0.511, *P* < 0.0001) and CIMT (adjusted R^2^ = 0.445, *P* < 0.0001), independently of traditional and emergent cardiovascular risk factors. Moreover, both constituted protective factors against subclinical atherosclerosis [OR: 0.993 (*P* = 0.002) and 0.231 (*P* = 0.025), respectively]. Receiver operating characteristic analysis pointed to the utility of serum Klotho (area under the curve [AUC]: 0.817, 95% CI: 0.736–0.898, *P* < 0.001) and its gene expression in PBCs (AUC: 0.742, 95% CI: 0.647–0.836, *P* < 0.001) to distinguish subclinical atherosclerosis. The reductions in serum and PBCs expression levels of Klotho in CKD patients are independently associated with the presence of for subclinical atherosclerosis. Further research exploring whether therapeutic approaches to maintain or elevate Klotho could reduce the impact of CVD in CKD patients is warranted.

Patients with chronic kidney disease (CKD) present high rates of morbidity and mortality, mainly of cardiovascular (CV) origin. In fact, CKD patients are more likely to die from cardiovascular disease (CVD) than to develop end-stage renal failure^1^. Different pathological mechanisms account for this higher risk of mortality including atherosclerosis, arterial stiffness, vascular calcification, congestive cardiomyopathy, capillary/myocyte mismatch in the heart, and sudden cardiac death^2^.

Although the involvement of accelerated atherosclerosis in the CV mortality observed in CKD patients has recently been questioned^3–7^, many clinical trials demonstrate that treatments aiming to reduce lipid burden are effective in reducing CV events, mainly in the early stages of CKD^8,9^. Additionally, several studies have reported an increase in the incidence and severity of coronary heart disease as the GFR decreases^10–12^ and the prevalence of atheromatous plaques in asymptomatic CKD patients throughout all stages of CKD^13^.

Nowadays, atherosclerosis is recognized as an inflammatory disorder and a wide range of inflammatory molecules and pathways are involved in all stages of the atherosclerotic process, from endothelial dysfunction to plaque rupture and thrombosis^14^. In CKD patients, not only inflammation, but also mineral bone metabolism derangements, have been associated with the appearing of CVD^15^. The *Klotho* gene is an important regulator of the mineral metabolism which is predominantly expressed in the distal tubular epithelial cells of the kidneys and, at lesser extent, in parathyroid glands, choroid plexus of the brain, vascular tissue, and peripheral blood cells (PBCs)^3–5^. There are two forms of Klotho, including a single-pass transmembrane protein and a soluble form generated from proteolytic cleavage of the extracellular domain of the membrane-bound form^6^. Soluble Klotho can be found in the cerebrospinal fluid, urine, and blood, and declines in CKD patients with the progression of the disease.

Importantly, experimental models point to the existence of Klotho protective effects upon vascular system that include the maintaining of endothelial wall homeostasis and the promotion of vascular health^7,16^, whereas Klotho deficiency triggers endothelial dysfunction and vascular calcification^17,18^. Moreover, several clinical studies also suggest that low serum levels of Klotho are associated with the prevalence and severity of CVD^19–21^ and all-cause mortality^22^, being associated with markers of vascular dysfunction and with the incidence of atherosclerosis^10,23^. Moreover, lower soluble Klotho level has been proposed as a newly predictor of atherosclerosis, being associated with increased epicardial fat thickness and carotid intima-media thickness (CIMT) and with decreased flow-mediated dilation in healthy subjects^24^. These associations are particularly relevant in kidney disease patients since the largest proportion of systemic Klotho is generated by the kidneys and its levels are reduced in all stages of the disease^11^. Thus, the decrease in Klotho serum levels has been described as an independent risk factor for coronary artery calcification in patients with maintenance hemodialysis^25^.

Therefore, Klotho has been suggested as a master regulator of CVD, with a potential role in the pathogenesis of atherosclerosis in CKD patients. However, whether serum and PBCs gene expression levels of Klotho are also related to early markers of subclinical atherosclerotis in patients with CKD is unknown. To address this hypothesis, we conducted a single-center cross-sectional study in patients with moderate to severe CKD, determining serum and PBCs gene expression levels of this protein and their independent relationship with two surrogate markers of subclinical atherosclerosis: ankle-brachial index (ABI) and CIMT. We also determined serum and PBCs expression levels of three inflammatory cytokines involved in the atherosclerotic process: tumor necrosis factor α (TNFα) and the interleukins (IL) 6 and 10.

## Methods

### Patients

A cross-sectional single-center study was conducted recruiting patients from the Nephrology Service of the University Hospital Nuestra Señora de Candelaria (Santa Cruz de Tenerife, Spain). From January-December 2006, 387 patients were initially evaluated and 103 were finally enrolled in the study. Inclusion criteria included CKD patients in stages 3–4 (estimated glomerular filtration rate (eGFR) 15–60 ml/min/1.73 m^2^ according to the Modification of Diet in Renal Disease Study-4 (MDRD-4) equation), who were older than 18 years of age, and who did not have history of known atherosclerotic cardiovascular disease. Exclusion criteria included history of heart failure; chronic inflammatory, immunologic, or tumoral disease; positive serology to hepatitis B, hepatitis C, or HIV; acute inflammatory or infectious intercurrent episodes in the previous month; institutionalization; receipt of immunotherapy or immunosuppressive treatment; and inability or unwillingness to provide informed consent. Patients with ABI values ≥ 1.3 were also excluded. The study protocol was approved by the Institutional Ethics Committee of the University Hospital Nuestra Señora de Candelaria and complied with ethical standards of the Declaration of Helsinki. Written informed consent was obtained from all participants.

### Biochemical parameters measurements and vascular assessments

All samples were drawn in the morning after 8 h of fasting. After centrifugation, serum samples were aliquoted and immediately frozen at − 80 °C. Routine biochemical parameters were measured using standard methods. Concentrations of serum Klotho protein were measured by a solid phase sandwich ELISA using the human soluble α-Klotho assay kit (Immuno-Biological Laboratories, Takasaki, Japan) according to manufacturer’s instructions. The assay sensitivity was 6.15 pg/mL and the intra and inter-assay coefficients of variation were 2.7–3.5% and 2.9–11.4%, respectively. The serum levels of the inflammatory cytokines TNFα, IL6, and IL10 were measured by ELISA methods (Quantikine®, R&D Systems, Abingdon, UK). Minimum detectable concentrations were 0.10 pg/mL, 0.70 pg/mL, and 0.09 pg/mL, respectively. Intra- and inter-assay coefficients of variability were < 10.8%. Serum Klotho, TNFα, IL6, and IL10 levels were expressed as pg/mL. High-sensitivity serum C-reactive protein (hsCRP) was measured by a high-sensitivity particle enhanced immunoturbidimetric fully automated assay (Roche Diagnostics GmbH, Mannheim, Germany) in a Cobas 6000 analyzer from the same manufacturer with a sensitivity of 0.3 mg/L and intra- and inter-assay coefficients of variation of 1.6% and 8.4%, respectively. hsCRP levels were expressed as mg/L.

The assessment of subclinical atherosclerotic disease was made by measurement of ABI and CIMT. The ABI was calculated using a portable pulse detector (Ultrasonic Mini Doppler ES-100VX; Hayashi Denki Co., Ltd., Kawasaki, Japan) with an 8 mHz probe. Measurements of CIMT was performed by a unique reader in a blinded fashion by ultrasonography of the carotid arteries with a high-resolution ultrasound (Philips ATL 5000 HDI, Royal Philips Electronics, Amsterdam, The Netherlands) equipped with a 6–13 MHz linear array transducer. We defined patients having subclinical atherosclerosis as those having ABI < 0.9 and/or CIMT ≥ 0.9 mm, according to the Guidelines for the management of arterial hypertension released by the Task Force for the Management of Arterial Hypertension of the European Society of Hypertension (ESH) and the European Society of Cardiology (ESC)^26^.

### Gene expression analysis

For analysis of gene expression in PBCs, 2.5 ml samples of whole-blood were collected in PAXgene blood RNA tubes (BD, Franklin Lakes, NJ) at the same time as serum samples. Total RNA was isolated from these tubes using a PAXgene blood RNA kit (Qiagen, Valencia, CA) according to manufacturer’s specifications and quantified using a Thermo Scientific NanoDrop Lite Spectrophotometer (Thermo Fisher Scientific, MA, USA). cDNA was obtained using a High-Capacity RNA-to-cDNA kit (Thermo Fisher Scientific, Foster City, CA, USA) for further analysis. Transcripts of Klotho gene (*KL*), *TNF*, *IL6*, *IL10,* and glyceraldehyde-3-phosphate dehydrogenase (*GAPDH*) as constitutive gene, were measured by real-time TaqMan quantitative PCR (qRT-PCR) with TaqMan Fast Universal PCR master mix (Thermo Fisher Scientific) in a 7500 Fast Real-Time PCR System (Thermo Fisher Scientific). TaqMan gene expression assays for each transcript were: Hs00183100_m1 [*KL*], Hs00174128_ml [*TNF*], Hs00985639_ml [*IL6*], Hs0961622_m1 [*IL10*], and Hs99999905_m1 [*GAPDH*]. The level of target mRNA was estimated by relative quantification using the comparative method (2^−ΔΔCt^) by normalizing to *GAPDH* expression. mRNA levels were expressed as arbitrary units (a.u.). Quantification of each cDNA sample was tested in triplicate. A corresponding non-reverse transcriptase reaction was included as a control for DNA contamination.

### Statistical analysis

Continuous variables were checked for the normal distribution assumption using the Kolmogorov–Smirnov statistic. Non-normally distributed variables were expressed as the median (interquartile range) and normally distributed variables were expressed as the mean ± SD. Categorical data were expressed as number and percent frequency. Differences between groups were analyzed using Chi-square test, Student’s t-test or the Mann–Whitney U-test as appropriate. The Spearman rank correlation was used to determine the correlations between two variables. Backward stepwise multiple regression analysis was performed to determine the independent association between patient clinical parameters as potential predictor variables (age, sex, body mass index (BMI), hypertension (HT), diabetes mellitus (DM), smoking, dyslipidemia, serum uric acid, eGFR, urinary albumin excretion (UAE), phosphorus, hsCRP, serum and PBCs expression levels of TNFα, IL6, and IL10, and Klotho) and ABI and CIMT values as dependent variables. Tolerance and variance inflation factor were analyzed in order to exclude collinearity. A multiple logistic regression was performed to assess independent predictors of the presence of subclinical atherosclerosis. For this purpose, we adopted three models: in model 1, we introduced age, HT, smoking, DM, dyslipidemia, macroalbuminuria and eGFR. In model 2, we additionally included the serum and blood expression levels of IL6 and of the ratio TNFα/IL10. Finally, in model 3 we adjusted the analysis for the serum and PBCs expression levels of Klotho. Area under the curve (AUC) from receiver operating characteristic (ROC) analysis was performed to examine the ability of of serum and PBCs expression levels of Klotho in distinguishing between patients with and without subclinical atherosclerosis and to identify the optimal cut-off values. A value of *P* < 0.05 was considered to be statistically significant. All analyses were performed using SPSS software version 25 (IBM Corp. Armonk, NY, USA).

## Results

### Characteristics of the patients and biochemical parameters

The characteristics of the study population including demographic and laboratory data are shown in Table 1. A total of 103 patients with CKD (52 males; mean age 67.3 ± 7.9 years) with a median eGFR of 38.63 (35.5–44) ml/min/1.73m^2^ were included. The median and interquartile range was 618.3 (492.9–785.4) pg/mL for serum Klotho concentrations and 1.9 (1.6–2.6) a.u. for *Klotho* mRNA levels in PBCs.

**Table 1:** Clinical characteristics.

	No subclinical atherosclerosis	Subclinical atherosclerosis	*P* value
**Characteristics**			
N	59	44	
Age (years)	67 ± 7.8	67.8 ± 8.02	0.808
Male (%)	30 (50.8)	22 (50)	0.619
Systolic BP (mm Hg)	140 (138–152)	140 (134–148)	0.398
Diastolic BP (mm Hg)	88 (82–90)	88 (82–90)	0.864
Body mass index (kg/m^2^)	29 (28–31)	29 (25.3–31)	0.941
ABI	1.05 (0.97–1.10)	0.87 (0.83–0.89)	< 0.001
CIMT (mm)	0.73 ± 0.066	0.80 ± 0.096	< 0.001
**Comorbidities**			
Diabetes mellitus (%)	18 (30.5)	23 (52.3)	< 0.05
Hypertension (%)	53 (89.8)	42 (95.5)	0.291
Current smokers (%)	14 (23.7)	22 (50)	< 0.01
Dyslipidemia (%)	44 (74.6)	33 (75)	0.961
Macroalbuminuria (%)	24 (40.7)	35 (79.5)	< 0.001
**Laboratory data**			
Hemoglobin (g/dl)	11.8 (11.2–12.1)	11.9 (11.5–12.4)	0.203
Creatinine (mg/dl)	1.9 (1.38–2.8)	1.87 (1.54–2.39)	0.602
eGFR (ml/min/1.73 m2)	38.63 (36.1–47.8)	38.6 (34.4–42.4)	0.277
UAE (mg/g)	214 (132–600)	979 (359–1277)	< 0.001
Albumin (g/dL)	3.9 (3.5–4)	3.74 (3.5–3.95)	0.347
Total cholesterol (mg/dL)	179 (149–193)	161 (153–201)	0.875
HDL-C (mg/dL)	42 (37–46)	42 (36–46)	0.625
LDL-C (mg/dL)	92 (80–109)	103 (80.3–115.8)	0.259
Triglycerides (mg/dL)	157 ± 39.6	143.1 ± 41.9	0.084
Uric acid (mg/dL)	6.63 ± 1.59	6.61 ± 1.3	0.942
Glucose (mg/dL)	79 (59–106)	108 (83.3–143.8)	< 0.05
Calcium (mg/dL)	9.2 (8.9–9.8)	9.3 (8.9–9.8)	0.896
Phosphorus (mg/dL)	4.1 (3.8–5)	4.2 (3.9–4.8)	0.928
Klotho (pg/mL)	744.92 (591.05–841.2)	523.96 (361–635.6)	< 0.001
hs-CRP (mg/L)	5.29 (3.3–7)	5.34 (3.43–7.3)	0.936
TNFα (pg/mL)	15.6 (12–17.9)	14.9 (11.9–17.6)	0.844
IL6 (pg/mL)	3.2 (2.4–4.9)	7.1 (3.9–9.4)	< 0.001
IL10 (pg/mL)	36.8 (24–50.3)	35 (29.4–42.4)	0.844
*KL* mRNA (a.u.)	2.2 (1.8–3)	1.7 (1.5–2.1)	< 0.001
*TNF* mRNA (a.u.)	1.9 (1.6–2.7)	1.8 (1.29–2.3)	0.107
*IL6* mRNA (a.u.)	1.8 (1.5–2.2)	1.8 (1.5–2.3)	0.970
*IL10* mRNA (a.u.)	1.25 (1.11–1.37)	1.27 (1.06–1.38)	0.896

Biochemical assessments and gene expression analysis according to the presence of subclinical cardiovascular disease.

*BP* blood pressure, *ABI* ankle-brachial index, *CIMT* carotid intima-media thickness, *eGFR* estimated glomerular filtrate rate, *UAE* urinary albumin excretion, *HDL-C* high-density lipoprotein cholesterol, *LDL-C* low-density lipoprotein cholesterol, *hs-CRP* high sensitivity C-reactive protein, *TNFα* tumor necrosis factor, *IL* interleukin *KL* Klotho gene.

Forty-four of patients (42.7%) presented subclinical atherosclerosis: 41 patients had ABI < 0.9 and 10 had CIMT ≥ 0.9 mmWe compared the differences between this group of patients to those without subclinical atherosclerosis. The prevalence of patients with DM (52.3 vs. 30.5%; *P* < 0.05), macroalbuminuria (79.5 vs. 40.7; *P* < 0.001), and smokers (50 vs. 23.7%; *P* < 0.01) was higher in this group. No differences were observed in age, sex, BMI, HT, or dyslipidemia. Regarding laboratory data, CKD patients with subclinical atherosclerosis presented higher levels of glucose, UAE, and IL6, and reduced serum concentrations of Klotho as well as lower mRNA expression levels in PBCs. No differences were observed for the rest of inflammatory parameters according to the presence of subclinical atherosclerosis, including serum levels and expression in PBCs of TNFα and IL10, and the expression of *IL6* in PBCs. Similarly, there were no differences in Klotho levels, neither soluble or PBCs expression, in subjects according to the presence of HT or dyslipidemia. Serum Klotho concentrations were lower in patients with DM and in smokers. Macroalbuminuric patients presented lower levels of both serum and PBCs expression of Klotho.

The characteristics of subjects stratified by tertiles of soluble and PBCs gene expression levels of Klotho are shown in Table 2. CKD patients with higher Klotho (tertiles 3), presented significantly higher ABI values (*P* < 0.001 for both Klotho determinations) and reduced CIMT (*P* < 0.001 for serum levels and *P* < 0.01 for *KL* expression in PBCs), which resulted in a lower prevalence of subclinical atherosclerosis (*P* < 0.001 for both determinations of Klotho). The prevalence of smokers and macroalbuminuric patients were lower in the higher tertiles (*P* < 0.05 and *P* < 0.01, respectively for both determinations of Klotho). Regarding laboratory data, patients in the upper tertiles of Klotho levels presented higher eGFR values. This difference was significant for *KL* expression in PBCs (*P* < 0.001) and almost significant for serum Klotho levels (*P* = 0.096). UAE was significantly reduced in higher tertiles of serum (*P* < 0.001) and PBCs gene expression (*P* < 0.01) levels of Klotho. Uric acid concentration was also reduced in subjects in the higher tertiles of serum Klotho (*P* < 0.05). Regarding inflammatory cytokines, serum IL6 levels were significantly reduced in higher tertiles of both serum and PBCs gene expression levels of Klotho (*P* < 0.001). Similarly, PBCs expression levels of *TNF* were also lower in higher tertiles of PBCs mRNA *KL* levels (*P* < 0.05).

**Table 2:** Clinical characteristics and general biochemical assessments stratified by tertiles of serum Klotho (pg/mL) (A) or PBCs *KL* mRNA (a.u.) (B) levels.

A	Tertile 1 < 559 pg/mL	Tertile 2 559–730 pg/mL	Tertile 3 > 730 pg/mL	*P* value for trend
**Characteristics**				
N	34	35	34	–
Age (years)	69.9 ± 6.8	64.9 ± 7.5	67.2 ± 8.6	0.031
Male (%)	16 (47)	18 (51.4)	15 (44.1)	0.635
Systolic BP (mm Hg)	140 (134–148)	140 (138–152)	140 (135.8–148.5)	0.356
Diastolic BP (mm Hg)	90 (82–90)	88 (82–90)	85.5 (74–90.5)	0.32
Body mass index (kg/m^2^)	29 (26–31)	28 (25–31)	29.5 (28–31.3)	0.292
ABI	0.89 (0.84–0.94)	0.92 (0.87–1)	1.06 (0.99–1.13)	< 0.001
CIMT (mm)	0.816 ± 0.092	0.758 ± 0.077	0.71 ± 0.065	< 0.001
**Comorbidities**				
sCVD (%)	25 (73.5)	17 (48.6)	2 (0.06)	< 0.001
Diabetes mellitus (%)	16 (47.1)	15 (42.9)	10 (29.4)	0.371
Hypertension (%)	32 (94.1)	34 (97.1)	29 (85.3)	0.218
Current smokers (%)	16 (47.1)	14 (0.4)	6 (17.6)	< 0.05
Dyslipidaemia (%)	25 (73.5)	25 (71.4)	27 (77.1)	0.235
Macroalbuminuria (%)	25 (73.5)	22 (62.9)	12 (35.3)	< 0.01
**Laboratory data**				
Hemoglobin (g/dl)	11.8 (11.3–12.1)	12 (11.4–12.4)	11.8 (11.3–12.3)	0.703
Creatinine (mg/dl)	1.87 (1.49–2.61)	1.8 (1.4–2.8)	1.9 (1.48–2.68)	0.542
eGFR (ml/min/1.73 m^2^)	36.7 (34.1–39.5)	39.4 (35.4–43.1)	38.62 (36.5–51.92)	0.096
UAE (mg/g)	1007 (238–1210)	359 (190–1240)	180 (117.3–534.5)	< 0.001
Albumin (g/dL)	3.7 (3.5–4)	3.7 (3.4–3.9)	3.9 (3.7–4)	0.169
T-cholesterol (mg/dL)	181 (155–201)	156 (142–193)	179 (152–190)	0.359
HDL-C (mg/dL)	42 (38–46)	42 (36–45)	41.5 (34.5–46)	0.507
Triglycerides (mg/dL)	148.6 ± 35.9	143.3 ± 39.2	161.7 ± 46.2	0.267
Uric acid (mg/dL)	7.1 ± 7.2	6.21 ± 1.49	6.56 ± 1.51	< 0.05
Glucose (mg/dL)	103.5 (85.2–145)	99 (79–140)	83.5 (75–120)	0.064
Calcium (mg/dL)	9.2 (8.9–9.7)	9.3 (9–10)	9.34 (8.98–9.8)	0.562
Phosphorus (mg/dL)	4.3 (3.8–4.9)	4.2 (3.8–4.9)	4.1 (3.8–5)	0.974
hs-CRP (mg/L)	6 (3.4–7.5)	4.87 (3.21–6.8)	6.23 (3.43–7.26)	0.283
TNFα (pg/mL)	15.9 (10.9–17.3)	15.6 (12.8–18.4)	15.1 (11.8–18.3)	0.764
IL6 (pg/mL)	7.1 (3.9–8.9)	4.4 (2.5–9)	3.1 (2.28–3.9)	< 0.001
IL10 (pg/mL)	35 (29.1–40.7)	35.6 (25–48.3)	38.4 (23.1–55.4)	0.644
*TNF* mRNA (a.u.)	1.9 (1.5–2.63)	1.8 (1.4–2.3)	1.95 (1.6–2.9)	0.115
*IL6* mRNA (a.u.)	1.9 (1.6–2.83)	1.9 (1.5–2.3)	1.75 (1.5–2.12)	0.525
*IL10* mRNA (a.u.)	1.29 (1.14–1.39)	1.27 (1.06–1.39)	1.235 (1.1–1.34)	0.284

B	Tertile 1 < 1.7 a.u	Tertile 2 1.7–2.3 a.u	Tertile 3 > 2.3 a.u	*P* value for trend
**Characteristics**				
N	32	36	35	–
Age (years)	67.6 ± 7.8	69.5 ± 7.3	64.9 ± 8	0.064
Male (%)	14 (43.8)	18 (50)	20 (57.5)	0.427
Systolic BP (mm Hg)	140 (134–148)	140 (138–149)	140 (136–152)	0.451
Diastolic BP (mm Hg)	87 (82–91.5)	89 (82–90)	88 (82–92)	0.912
Body mass index (kg/m^2^)	28.5 (26–31)	29.5 (26.3–31)	29 (26–31)	0.894
ABI	0.89 (0.86–0.99)	0.905 (0.873–0.967)	1.06 (0.97–1.14)	< 0.001
CIMT (mm)	0.799 ± 0.094	0.777 ± 0.079	0.72 ± 0.073	< 0.01
**Comorbidities**				
sCVD (%)	20 (62.5)	18 (50)	6 (45.7)	< 0.001
Diabetes mellitus (%)	12 (37.5)	16 (44.4)	13 (37.1)	0.319
Hypertension (%)	29 (90.6)	34 (94.4)	32 (91.4)	0.223
Current smokers (%)	14 (43.8)	18 (50)	4 (11.4)	< 0.05
Dyslipidaemia (%)	26 (81.3)	25 (69.4)	26 (74.3)	0.314
Macroalbuminuria (%)	26 (81.3)	20 (55.6)	13 (37.1)	< 0.01
**Laboratory data**				
Hemoglobin (g/dl)	11.8 (11.6–12.5)	11.8 (11.4–12.2)	11.8 (11.2–12.1)	0.557
Creatinine (mg/dl)	2.1 (1.5–2.6)	1.88 (1.42–2.65)	1.64 (1.4–2.8)	0.742
eGFR (ml/min/1.73 m^2^)	36.7 (34.3–41)	38.6 (35–39.6)	42.3 (38–54)	< 0.001
UAE (mg/g)	800 (323–1185)	395 (180.5–1200)	214 (120–450)	< 0.01
Albumin (g/dL)	3.7 (3.5–3.9)	.8 (3.6–4)	3.9 (3.4–4)	0.212
T-cholesterol (mg/dL)	179 (155–197)	165 (146.5–184)	180 (155–196)	0.518
HDL-C (mg/dL)	41.5 (35–42)	42 (39–46)	42 (37–46)	0.177
Triglycerides (mg/dL)	150.4 ± 30.6	151 (120.3–184.3)	145 (124–180)	0.88
Uric acid (mg/dL)	6.5 ± 1.44	6.74 ± 1.6	6.57 ± 1.39	0.778
Glucose (mg/dL)	92.5 (79.5–127)	96 (81–140.8)	87 (77–138)	0.613
Calcium (mg/dL)	9.1 (8.8–9.6)	9.2 (9–9.8)	9.4 (9–9.8)	0.293
Phosphorus (mg/dL)	4.4 (3.9–5)	4.2 (3.9–4.9)	7 (6.6–7.4)	0.244
hs-CRP (mg/L)	5.75 (3.92–7.32)	5.12 (3–7.3)	5.3 (3.2–7.1)	0.9
TNFα (pg/mL)	13.1 (10.9–17.7)	15.2 (12.7–16.3)	16.4 (12–19.7)	0.119
IL6 (pg/mL)	6.4 (3.6–9.3)	4.8 (3.43–7.78)	2.9 (2.4–3.9)	< 0.001
IL10 (pg/mL)	30.9 (23.2–42.3)	38.2 (33–46.4)	36.1 (24.7–54)	0.115
*TNF* mRNA (a.u.)	1.8 (1.2–2.6)	1.8 (1.5–2.1)	2.1 (1.8–3)	< 0.05
*IL6* mRNA (a.u.)	1.95 (1.5–2.78)	1.8 (1.6–2.2)	1.8 (1.5–2.2)	0.339
*IL10* mRNA (a.u.)	1.26 (1.2–1.4)	1.3 (1.1–1.4)	1.25 (1.14–1.39)	0.802

*BP* blood pressure, *ABI* ankle-brachial index, *CIMT* carotid intima-media thickness, *sCVD* subclinical cardiovascular disease, *eGFR* estimated glomerular filtrate rate, *UAE* urinary albumin excretion, *T-cholesterol* total cholesterol, *HDL-C* high-density lipoprotein cholesterol, *hs-CRP* high sensitivity C-reactive protein, *TNFα* tumor necrosis factor, *IL* interleukin.

### Correlations and multivariate analysis

Serum and PBCs mRNA levels of Klotho were significantly correlated (r = 0.346, *P* < 0.0001). We found a trend for an inverse correlation between age and serum Klotho levels (r = − 0.177, *P* = 0.073). Serum Klotho levels were directly and significantly correlated with eGFR (r = 0.219, *P* = 0.026) and inversely correlated with UAE (r = − 0.455, *P* < 0.0001), glucose (r = − 0.266, *P* = 0.007), and serum levels of IL6 (r = − 0.515, *P* < 0.0001). PBCs mRNA *KL* levels were also directly and significantly correlated with eGFR (r = 0.333, *P* < 0.001), and with both serum and PBCs mRNA levels of TNFα (r = 0.199, *P* = 0.044; r = 0.227, *P* = 0.021, respectively), and inversely correlated with UAE (r = − 0.387, *P* < 0.0001), serum IL6 levels (r = − 0.455, *P* < 0.0001).

Regarding subclinical atherosclerosis markers, both serum and PBCs mRNA Klotho levels were positively correlated with ABI (r = 0.556, *P* < 0.0001 and r = 0.373, *P* < 0.0001, respectively) and inversely correlated with CIMT (r = − 0.541, *P* < 0.0001 and r = − 0.437, *P* < 0.0001, respectively). Among inflammatory markers, only serum IL6 levels presented significant associations with subclinical atherosclerosis, being inversely related with ABI (r = − 0.568, *P* < 0.0001) and positively related with CIMT (r = 0.558, *P* < 0.0001). PBCs expression of *TNF* only correlated with ABI (r = 0.244, *P* = 0.013) and with UAE (r = − 0.29, *P* = 0.003).

To test the independent association between the levels of soluble and PBCs expression of Klotho and the two markers of atherosclerosis, backward stepwise multiple regression analysis was performed with ABI and CIMT as dependent variables. The results showed that serum and blood mRNA Klotho levels, together with serum IL6, were positively related and significantly associated with the values of ABI (adjusted R^2^ = 0.537, *P* < 0.0001) and CIMT (adjusted R^2^ = 0.37, *P* < 0.0001) (Table 3). Collinearity was assessed by examining tolerance and the variance inflation factor (VIF) for each variable in both regression analysis. Tolerance and VIF values were higher than 0.60 and lower than 1.5 for all variables in any of the analysis. Therefore, collinearity was excluded.

**Table 3:** Multiple backward stepwise regression analysis for ABI and CIMT as dependent variables.

	Adjusted R^2^	β	Standard error	t	*P*
**ABI**	0.537				< 0.0001
Current smokers		− 0.206	0.019	− 2.735	0.0075
Diabetes mellitus		− 0.163	0.017	− 2.342	0.0212
Serum IL6 (pg/mL)		− 0.288	0.003	− 3.454	0.00083
Serum Klotho (pg/mL)		0.236	0.000052	2.841	0.0055
PBCs *KL* mRNA (a.u.)		0.238	0.013	2.963	0.0037
**CIMT**	0.370				< 0.0001
Serum IL6 (pg/mL)		0.229	0.139	2.391	0.019
Serum Klotho (pg/mL)		− 0.366	0.000238	− 3.887	< 0.001
PBCs *KL* mRNA (a.u.)		− 0.171	0.0595	− 1.885	0.041

*ABI* ankle-brachial index, *CIMT* carotid intima-media thickness, *PBCs* peripheral blood cells, *IL* interleukin, *KL* Klotho gene.

**Figure 1:**
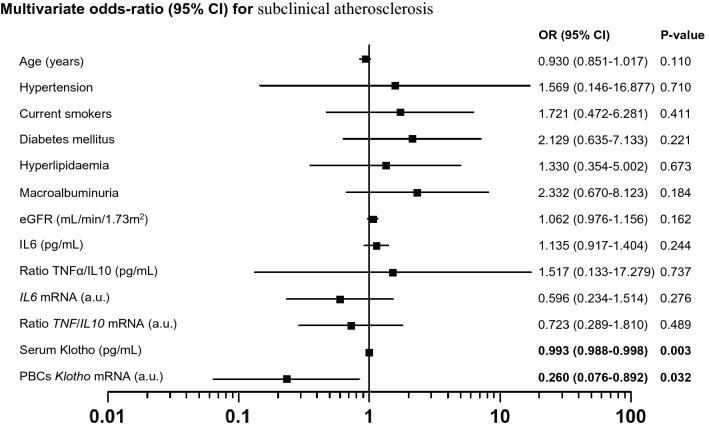
Multivariate odds ratio for subclinical atherosclerosis displayed as the odds ratio (OR) with 95% confidence intervals (CIs).

The multivariate logistic regression modelling results, using the presence/absence of subclinical atherosclerosis as the dependent variable, are presented in Table 4. Traditional risk factors for CVD (age, HT, smoking, DM, and dyslipidemia) were entered as covariates (model 1), with additional models in which markers of renal function (eGFR and macroalbuminuria) (model 2), and inflammatory cytokines (model 3) were added. Results of this analyses showed that both serum and PBCs mRNA levels of Klotho were covariates associated with subclinical atherosclerosis, indicating that the levels of both variables are protective factors for the presence of this condition in CKD patients in stage 3–4 (Table 4 and Fig. 1). The results were similar when analyzing these variables separately.

**Table 4:** Multivariate logistic regression analysis for the presence of subclinical atherosclerosis with serum Klotho and *KL* PBCs mRNA levels as independent variables.

	Unadjusted		Model 1		Model 2		Model 3	
	OR (95% CI)	*P*-value	OR (95% CI)	*P*-value	OR (95% CI)	*P*-value	OR (95% CI)	*P*-value
Serum Klotho (pg/mL)	0.993 (0.99–0.996)	< 0.001	0.992 (0.988–0.996)	< 0.001	0.992 (0.988–0.996)	< 0.001	0.993 (0.988–0.998)	0.003
PBCs *KL* mRNA (a.u.)	0.291 (0.12–0.707)	0.006	0.24 (0.086–0.673)	0.007	0.218 (0.069–0.694)	0.01	0.260 (0.076–0.892)	0.032

OR values are expressed per unit variation.

Model 1 was adjusted by age, hypertension, current smokers, diabetes mellitus, hyperlipidemia.

Model 2 was Model 1 adjusted by macroalbuminuria and eGFR.

Model 3 was Model 2 adjusted by serum levels of IL6 and TNFα/IL10 and PBCs mRNA levels of *IL6* and *TNF/IL10.*

*eGFR* estimated glomerular filtrate rate, *IL* interleukin, *TNFα* tumor necrosis factor.

### ROC curve analysis

**Figure 2:**
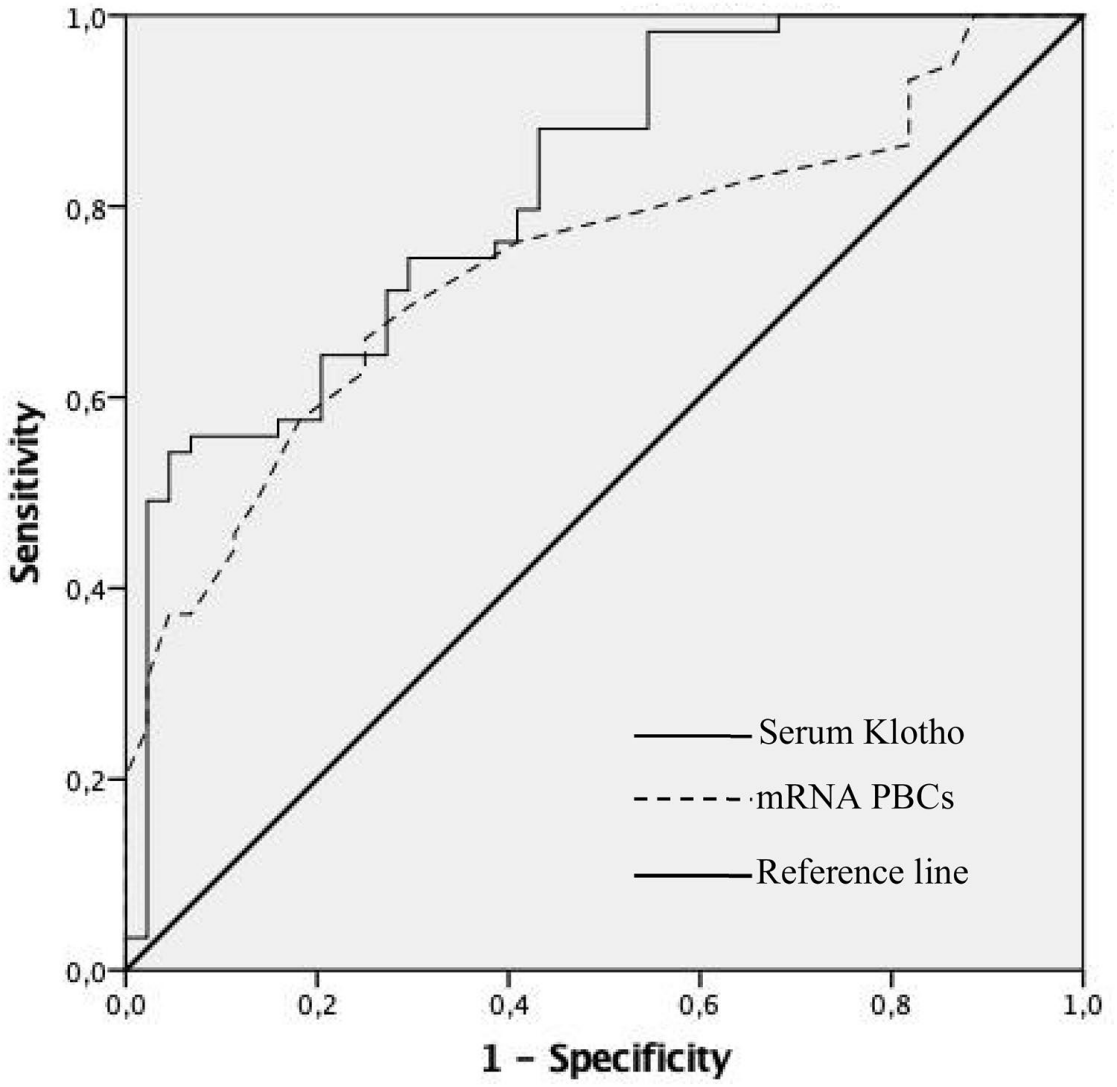
ROC curves of serum and mRNA PBCs levels of Klotho for distinguishing subclinical atherosclerosis.

The ROC curve analysis was used to determine the ability of the serum and PBCs gene expression levels of Klotho to distinguish between subjects according to the presence of subclinical atherosclerosis (Fig. 2). AUC for serum and PBCs expression levels of Klotho were 0.817 (95% CI: 0.736–0.898, *P* < 0.001) and 0.742 (95% CI: 0.647–0.836, *P* < 0.001), respectively. The optimal cut-off values for subclinical atherosclerosis were 553.04 pg/ml (specificity 56.8% and sensitivity 88.1%) for serum Klotho, and 2.05 a.u. (specificity 75% and sensitivity 62.7%) for mRNA *KL* expression in PBCs.

## Discussion

In this study, we show that CKD patients with subclinical atherosclerosis present reduced levels of serum and mRNA expression in PBCs of Klotho as compared with CKD patients without this clinical status. In addition, both Klotho variables are significantly associated with two markers of subclinical atherosclerosis, ABI and CIMT, independently of cardiovascular risk factors and inflammatory parameters. Specifically, both determinations directly correlated with ABI, whereas presented an inverse association with CIMT. Moreover, in multivariate analyses, both Klotho variables were found to be independent determinants of ABI and CIMT, and independent predictors of subclinical atherosclerosis. Whether reduction in serum and PBCs gene expression levels of Klotho directly promote or favor the progression of atherosclerosis in CKD is an intriguing question that requires further study.

Atherosclerosis is a major complication in CKD patients that dramatically increase CVD morbidity and mortality. In this study, we employed two widely used methods for assessing the presence of subclinical atherosclerosis: ABI and CIMT. ABI is a quick, noninvasive way to check peripheral artery disease, being considered as an indirect indicator of general atherosclerosis and an independent predictor of cardiovascular and all-cause mortality in CKD and hemodialysis (HD) patients^12,27^. CIMT, is an ultrasound based quantitative parameter widely used as a direct marker of atherosclerotic disease. We have chosen CIMT in this study to determine subclinical atherosclerotic disease due to its independent association with increased cardiovascular risk and impaired renal function^28^, being able to predict ischemic events and long-term mortality in patients with different stages of CKD^29^, and in predialysis^30^ and HD patients^31^. Our results pointed to Klotho as an independent determinant of ABI and CIMT, even after adjusting for age, gender, blood pressure, smoking, DM, dyslipidemia, and other factors. Additionally, both Klotho variables in our study were significant predictors of subclinical atherosclerosis in a full adjusted model that included important cofounders such as age, HT, smoking, eGFR, DM, dyslipidemia, macroalbuminuria, and the serum and PBCs expression levels of the cytokines IL6, TNFα, and IL10.

Previous clinical studies have suggested that Klotho might play a role in the pathogenesis of atherosclerotic disease. Genetic variants of *Klotho* have been associated with high CIMT in hypertensive patients^32^ and with the risk of early-onset occult coronary artery disease^33^. Low soluble Klotho levels have been related to CVD in cross-sectional clinical studies carried out in elderly subjects (> 65 years old)^22^, in subjects with preserved renal function subjected to angiography^20^, and in diabetic patients^21^. Similar results have been observed in patients in HD, where diminished levels of circulating Klotho were related to cardiovascular events and mortality^34^. However, studies about the relationship between Klotho and markers of subclinical atherosclerosis in CKD patients are scarce and none of them have considered the expression of Klotho in PBCs. A recent cross-sectional study suggested that decreased soluble Klotho levels in 114 CKD patients were associated with signs of arterial stiffness, determined by increased brachial-ankle pulse wave velocity (baPWV) (adjusted OR = 0.60, 95% CI = 0.39–0.98, *P* = 0.0075)^23^. Similarly, a prospective study carried out in 63 patients with CKD showed that patients with serum soluble Klotho levels in the lower quartile (< 309 pg/mL) had significantly higher cardiovascular and all-cause mortality rates (hazard ratio = 4.17, 95% CI = 1.29–13.48, *P* = 0.018)^35^.

Experimental studies point to the existence of a role of Klotho in the maintaining of the vascular-health. Klotho-deficient mice show increased vascular endothelial permeability, impaired endothelial-dependent vasodilatation, reduced excretion of nitric oxide metabolites, and impaired angiogenesis and vasculogenesis^3–7,16,17,36,37^. These dysfunctions were recovered after parabiosis with wild-type mice^16^. In that study, serum Klotho stimulated endothelium-derived NO production, which ameliorated endothelial dysfunction and prevented vascular remodeling^16^. This protective effect on vascular cells was also observed in vitro, where Klotho was able to protect endothelial and vascular smooth muscle cells from inflammation and oxidation, critical factors in the progression of atherosclerosis^38–41^. Klotho attenuated cellular apoptosis and senescence in human umbilical vein endothelial cells (HUVECS) via mitogen-activated kinase and extracellular signal-regulated kinase pathways^38^. In rat aortic smooth muscle cells, Klotho gene transfer downregulated Nox2 protein expression and intracellular superoxide production, and attenuated angiotensin II-induced superoxide production, reducing oxidative damage and apoptosis^41^. Finally, Klotho may have a role in the modulation of endothelial inflammation by reducing TNFα-induced expression of adhesion molecules and NF-kB activation^39^ and by inhibiting retinoic-acid-inducible gene-I induced expression of IL6 and IL8 in HUVECS^40^.

Results of our study are in line with these previous findings and extends the association between Klotho and CVD to the reduced expression of Klotho in PBCs. Macrophages, monocytes, lymphocytes, and other PBCs participate in the immune response with a central role in the development of the inflammatory response associated with the atherogenic process. This role ranges from a contribution to the low-grade systemic inflammation that accompanies cardiovascular disease (secretion of pro- or anti-inflammatory factors into the systemic circulation) to the resolution of the local response of the vascular wall (environmental signal transduction, uptake of LDL or oxLDL particles, engulfment of dead cells, secretion of inflammatory cytokines or pro-resolving molecules, etc.)^42,43^. Klotho has been previously detected in PBCs and marked reductions in Klotho expression in PBCs has been related with aging and with the development of pathologies with an inflammatory component including atherosclerosis^41,44,45^. Although there are few studies that delve into the mechanisms of action, the up-regulation of Klotho in these cells could be beneficial in antagonizing the progression of atherosclerotic lesions through anti-inflammatory effects. In this sense, the expression of Klotho in these cells has been related with the attenuation of lipopolysaccharide (LPS)-induced acute inflammation in macrophages and with the inactivation of NF-kB signaling and the promotion of M2 polarization in these cells, which infiltrates atheromatous plaques and develop a defensive and repair response to vascular damage^46,47^. Moreover, it has also been related with the suppression of the stress response of the Golgi apparatus and endoplasmic reticulum, the reduction of the levels of oxidant radicals and pro-inflammatory cytokines, as well as with an increase in the production of anti-inflammatory cytokines and the preservation of the immune function in senescent monocytes^48,49^. All these mechanisms play key roles in the inflammatory response exerted by PBCs in the atherosclerotic process and, therefore, make Klotho expression in these cells an interesting target in such scenario. The presence of Klotho in these cells could be considered, albeit indirectly, as a possible actor involved in the preservation of vascular function.

Although our study provides novel information about the relationship between Klotho and subclinical atherosclerosis in CKD patients, we acknowledge several limitations. First, serum concentrations of vitamin D, fibroblast growth factor-23, and parathyroid hormone -factors related to Klotho and calcium phosphate metabolism, with potential impact on atherosclerosis- were not measured in our study, and therefore a possible influence on the relationship between Klotho and CVD cannot be completely ruled out. Moreover, protein expression levels of Klotho in PBCs were not assessed in these patients and mRNA variations in *KL* expression may not reflect substantial modifications in protein levels. Second, the sample size was relatively small, which does not allow generalization of the results. Thirdly, although we accounted for confounding of traditional and CKD-related cardiovascular risk factors, a potential for uncontrolled or residual confounding that could affect our results could be plausible. Finally, given the cross-sectional design of the study, we can only demonstrate associations without definitive inferences on their direction or causality. Our observations only show a relationship with an already developed clinical scenario and they do not provide information about the independent implication of Klotho levels variations in the development and progression of the atherosclerotic lesion.

Nevertheless, our study presents some strengths that deserve to be highlighted: it was a population-based sample of adults; the presence and severity of subclinical atherosclerosis were carefully analyzed by two different markers of atherosclerosis; and data included conventional and CKD-related cardiovascular risk factors, including the serum levels and PBCs expression of inflammatory parameters.

## Conclusions

In conclusion, in the present study we determined the association between serum concentrations and PBCs Klotho expression levels with markers of subclinical atherosclerosis in CKD patients, while simultaneously explored the possible confounding effects caused by CKD-MBD and inflammation-related risk factors. Further experimental and clinical studies are warranted to confirm our findings, to explore the effects and relationships of Klotho on the cardiovascular system, to evaluate the role of Klotho as a potential novel biomarker of CVD, and to assess the effect of therapeutic strategies directed to increase Klotho levels on atherosclerotic disease in CKD.

## Supplementary Information

Supplementary Figure S1.

Supplementary Figure S2.

Supplementary Information.

## Abbreviations

CKD: Chronic kidney disease
CVD: Cardiovascular disease
PBCs: Peripheral blood cells
TNFα: Tumor necrosis factor α
IL: Interleukin
sCVD: Subclinical atherosclerotic cardiovascular disease
ABI: Ankle-brachial index
CIMT: Carotid intima-media thickness
eGFR: Estimated glomerular filtration rate
MDRD: Modification of diet in renal disease study
hsCRP: High-sensitivity serum C-reactive protein
GAPDH: Glyceraldehyde-3-phosphate dehydrogenase
BMI: Body mass index
HT: Hypertension
DM: Diabetes mellitus
ROC: Receiver operating characteristic curve
baPWV: Brachial-ankle pulse wave velocity
HUVECS: Umbilical vein endothelial cells
LPS: Lipopolysaccharide
