# Supplementary figures and images for: Klotho as a biomarker of subclinical atherosclerosis in patients with moderate to severe chronic kidney disease
Source: Sci Rep. 2021 Aug 5;11:15877. doi: 10.1038/s41598-021-95488-4 (PMC8342510; doi:10.1038/s41598-021-95488-4)

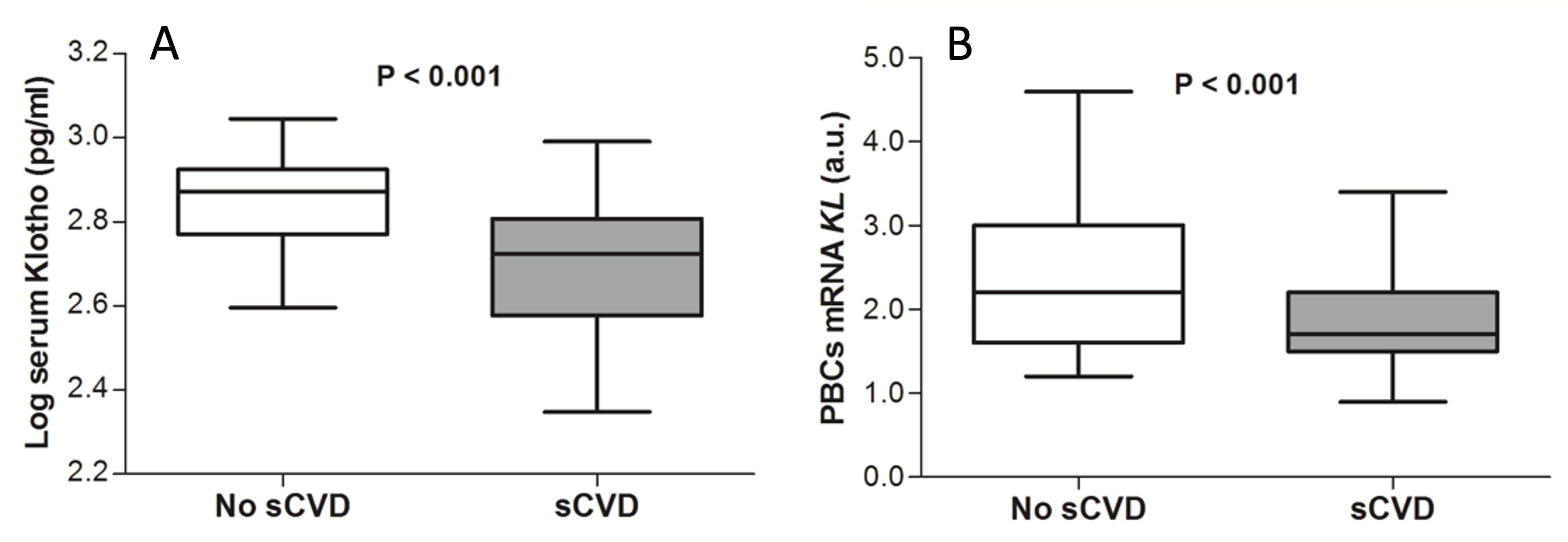

Supplement: Supplementary file 1 — Supplementary Figure S1. [file 41598_2021_95488_MOESM1_ESM.png]

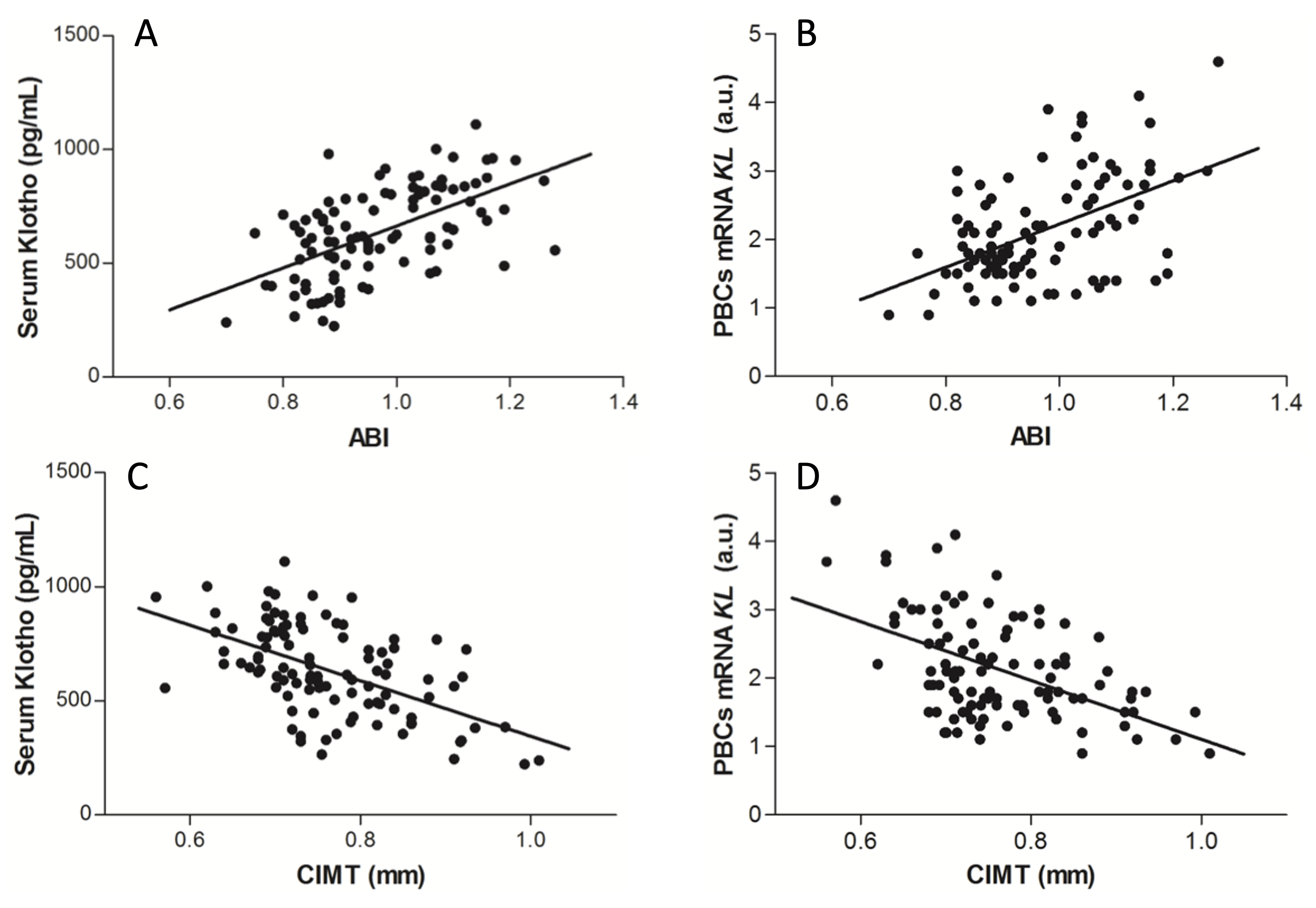

Supplement: Supplementary file 2 — Supplementary Figure S2. [file 41598_2021_95488_MOESM2_ESM.png]
