# Supplementary material for: Klotho as a biomarker of subclinical atherosclerosis in patients with moderate to severe chronic kidney disease
Source: Sci Rep. 2021 Aug 5;11:15877. doi: 10.1038/s41598-021-95488-4 (PMC8342510; doi:10.1038/s41598-021-95488-4)
Supplement: Supplementary file 3 — Supplementary Information. [file 41598_2021_95488_MOESM3_ESM.docx]

**Title page for supplementary material**

**Title: Klotho as a biomarker of subclinical atherosclerosis in patients with moderate to severe chronic kidney disease..**

Javier Donate-Correa PhD, Carla M. Ferri MSc, Ernesto Martín-Núñez MSc, Nayra Pérez-Delgado BPharm, Ainhoa González-Luis MSc, Carmen Mora-Fernández MD, Juan F. Navarro-González MD, PhD

**Figure legends:**

**Figure S1.** Differences in the log-transformed serum (A) and peripheral blood mRNA levels (B) of Klotho between CKD patients with and without subclinical atherosclerosis.

**Figure S2.** Correlations between serum and PBCs mRNA expression levels of Klotho and surrogate markers of atherosclerosis. Relationships between serum Klotho and ABI (A) and CIMT (C), and between PBCs gene expression of *KL* and ABI (B) and CIMT (D). N = 103.

| **Table S1.** Univariate correlation analysis between serum and gene expression levels Klotho and inflammatory cytokines and other factors. | | | | | | | | | | | | | | | | |
| --- | --- | --- | --- | --- | --- | --- | --- | --- | --- | --- | --- | --- | --- | --- | --- | --- |
|  | Serum levels | | | | | | | | Gene expression | | | | | | | |
|  | KL | | TNFα | | IL6 | | IL10 | | *KL* | | *TNF* | | *IL6* | | *IL10* | |
|  | r | *P* | r | *P* | R | *P* | r | *P* | r | *P* | r | *P* | r | *P* | r | *P* |
| Klotho (pg/mL) | 1 | - |  |  |  |  |  |  |  |  |  |  |  |  |  |  |
| TNFα (pg/mL) | 0.073 | 0.464 | 1 | - |  |  |  |  |  |  |  |  |  |  |  |  |
| IL6 (pg/mL) | **-0.515*** | **<0.0001** | 0.045 | 0.653 | 1 | - |  |  |  |  |  |  |  |  |  |  |
| IL10 (pg/mL) | 0.093 | 0.348 | -0.062 | 0.531 | 0.101 | 0.31 | 1 | - |  |  |  |  |  |  |  |  |
| *KL* mRNA (a.u.) | **0.346*** | **<0.0001** | **0.199*** | **0.044** | **-0.455*** | **<0.0001** | 0.172 | 0.083 | 1 | - |  |  |  |  |  |  |
| *TNF* mRNA (a.u.) | 0.074 | 0.46 | 0.16 | 0.107 | -0.144 | 0.148 | 0.023 | 0.816 | **0.227*** | **0.021** | 1 | - |  |  |  |  |
| *IL6* mRNA (a.u.) | -0.114 | 0.253 | 0.025 | 0.805 | 0.025 | 0.802 | 0.32 | 0.751 | -0.17 | 0.086 | -0.126 | 0.206 | 1 | - |  |  |
| *IL10* mRNA (a.u.) | 0.017 | 0.867 | 0.018 | 0.854 | -0.074 | 0.458 | **0.229*** | **0.02** | 0.087 | 0.377 | -0.002 | 0.988 | -0.125 | 0.209 | 1 | - |
| Age (years) | -0.177 | 0.073 | 0.062 | 0.534 | 0.107 | 0.281 | -0.069 | 0.49 | -0.114 | 0.203 | -0.092 | 0.358 | -0.004 | 0.969 | -0.038 | 0.706 |
| Body mass index (kg/m^2^) | 0.161 | 0,104 | -0.118 | 0.234 | 0.065 | 0.513 | 0.073 | 0.461 | 0.043 | 0.772 | -0.015 | 0.882 | 0.045 | 0.652 | 0.042 | 0.677 |
| ABI | **0.556*** | **<0.0001** | 0.017 | 0.866 | **-0.568*** | **<0.0001** | -0.017 | 0.865 | **0.373*** | **<0.0001** | **0.244*** | **0.013** | 0.019 | 0.848 | -0.01 | 0.919 |
| CIMT (mm) | **-0.541*** | **<0.0001** | -0.184 | 0.063 | **0.558*** | **<0.0001** | 0.08 | 0.42 | **-0.437*** | **<0.0001** | -0.024 | 0.811 | 0.094 | 0.347 | -0.029 | 0.775 |
| eGFR (mL/min/1.73m^2^) | **0.219*** | **0.026** | 0.096 | 0.332 | -0.178 | 0.073 | **0.438*** | **<0.0001** | **0.333*** | **<0.001** | 0.06 | 0.546 | **-0.203*** | **0.04** | 0.182 | 0.066 |
| UAE (mg/g) | **-0.455*** | **<0.0001** | -0.136 | 0.172 | **0.46*** | **<0.0001** | 0.089 | 0.371 | **-0.387*** | **<0.0001** | **-0.29*** | **0.003** | 0.06 | 0.546 | -0.042 | 0.672 |
| Glucose (mg/dL) | **-0.266*** | **0.007** | **-0.203*** | **0.04** | 0.156 | 0.115 | -0.012 | 0.9 | -0.101 | 0.285 | -0.138 | 0.164 | 0.003 | 0.977 | 0.043 | 0.666 |
| Total cholesterol (mg/dL) | -0.092 | 0.353 | 0.022 | 0.824 | 0.155 | 0.118 | 0.122 | 0.219 | 0.024 | 0.716 | 0.093 | 0.351 | 0.125 | 0.209 | -0.03 | 0.76 |
| HDL-C (mg/dL) | -0.102 | 0.307 | -0.005 | 0.96 | -0.025 | 0.804 | -0.023 | 0.821 | 0.026 | 0.72 | 0.174 | 0.079 | 0.057 | 0.569 | -0.068 | 0.496 |
| Calcium (mg/dL) | 0.1 | 0.316 | 0.137 | 0.168 | 0.044 | 0.659 | 0.189 | 0.056 | 0.173 | 0.081 | 0.154 | 0,121 | -0.18 | 0.068 | 0.149 | 0.132 |
| Phosphorous (mg/dL) | -0.024 | 0.813 | -0.135 | 0.175 | 0.013 | 0.896 | **-0.275*** | **0.005** | -0.159 | 0.1 | -0.124 | 0.211 | -0.002 | 0.981 | -0.079 | 0.428 |
| Creatinine (mg/dl) | 0.019 | 0.103 | 0.046 | 0.644 | 0.054 | 0.596 | **-0.46*** | **<0.0001** | -0.087 | 0.364 | -0.034 | 0.734 | 0.015 | 0.878 | -0.079 | 0.426 |
| Albumin (g/dL) | **0.199*** | **0.044** | 0.026 | 0.795 | 0.021 | 0.833 | **0.316*** | **0.001** | 0.188 | 0.057 | 0.064 | 0.52 | -0.036 | 0.716 | 0.192 | 0.052 |
| Uric acid (mg/dL) | -0.146 | 0.142 | -0.114 | 0.253 | -0.154 | 0,121 | -0.069 | 0.491 | 0.007 | 0.813 | 0.074 | 0.455 | 0.025 | 0.805 | 0.015 | 0.88 |
| hs-CRP (mg/L) | -0.054 | 0.591 | 0.189 | 0.056 | -0.027 | 0.787 | **-0.240*** | **0.015** | -0.044 | 0.704 | 0.06 | 0.55 | 0.041 | 0.682 | -0.155 | 0.118 |

Abbreviations: BP, blood pressure; ABI. ankle-brachial index; CIMT, carotid intima-media thickness; eGFR, estimated glomerular filtrate rate; UAE, urinary albumin excretion; HDL-C, high-density lipoprotein cholesterol; hs-CRP, high sensitivity C-reactive protein; TNFα, tumor necrosis factor; IL, interleukin; *KL*, Klotho gene.
